# Effects of soil fauna on litter decomposition in Chinese forests: a meta-analysis

**DOI:** 10.7717/peerj.12747

**Published:** 2022-01-10

**Authors:** Peng Zan, Zijun Mao, Tao Sun

**Affiliations:** 1 Northeast Forestry University, Key Laboratory of Forest Plant Ecology, Ministry of Education, Harbin, China; 2 Northeast Forestry University, College of Chemistry, Chemical Engineering and Resource Utilization, Harbin, China; 3 Chinese Academy of Sciences, Key Laboratory of Forest Ecology and Management, Institute of Applied Ecology, Shenyang, Liaoning province, China

**Keywords:** Climate, Litter quality, Cellulose, Nitrogen, Size effect

## Abstract

Litter quality and climate have been presumed to be the dominant factors regulating litter decomposition rates on broad spatial scales. However, the role of soil fauna on litter decomposition is poorly understood, despite the fact that it could strongly influence decomposition by fragmentation and subsequent modification of the activities of microorganisms.In this study, we carried out a meta-analysis on the effects of soil fauna on litter decomposition rates in Chinese forests, ranging from boreal to tropical forests, based on data from 20 studies. The effects of climatic factors on decomposition rate were assessed by comparing the contribution of soil fauna to litter decomposition from studies carried out at different latitudes.The degree of influence of the soil fauna was in the order tropical (200%) > subtropical (47%) > temperate forest (28%). Comparing the effect size of soil fauna, it was found that when soil fauna was excluded, the decomposition rate, calculated using Olson’s equation, was most affected in tropical forest (−0.77), while the litter decomposition rate both subtropical (−0.36) and temperate forest (−0.19) were also suppressed to varying degrees (*P* < 0.001). These results highlight that soil fauna could promote litter decomposition to different extents. Using stepwise multiple linear regression, the effect size of the soil fauna was negatively correlated with the cellulose and nitrogen concentrations of the initial litter material. In Chinese forests, litter decomposition rates were reduced, on average, by 65% when soil fauna was excluded. The impact of soil fauna on decomposition was shown to be closely related to climate and litter quality.

## Introduction

Litter decomposition drives most of the net primary production in terrestrial ecosystems (Aerts, 1997), so understanding how litter decomposes is important for predicting current and future carbon budgets (Aerts, 2006; Schlesinger & Bernhardt, 2013; Krishna & Mohan, 2017). Research suggests that litter decomposition is primarily controlled by climate, decomposers, and litter quality (Mori et al., 2020). Previous studies had shown that climate plays a major role in determining the speed of litter decomposition (Cornwell et al., 2008). The inclusion of soil fauna in a model will improve the predictive power of a regional or biological scale decomposition model (Wall et al., 2008). Globally, in terrestrial ecosystems, the climate and litter quality explain more than 65% of the variation in decomposition rate (García-Palacios et al., 2013). However, the contribution of soil biota to litter decomposition is not known.

The mechanisms through which soil fauna affect litter decomposition include (1) feeding on and fragmentation of litter (Read & Perez-Moreno, 2003; Moore et al., 2004), and (2) the effects of soil fauna activities on soil structure and microbial activities (Henning & Luxton, 1982; Anderson, Ineson & Huish, 1983; Maraun & Scheu, 1996; Castanho, Lorenzo & De Oliveira, 2012). For example, earthworms break up the litter by the act of consuming it, and their actions, including excretion, change the litter decomposition environment and subsequently stimulate microbial changes in the soil (Frouz, 2018). These factors will have various degrees of impact on decomposition rates. In addition, climate and litter quality also have indirect effects on soil fauna (Wardle, Walker & Bardgett, 2004). Heneghan et al. (1999) considered that the effect of soil fauna on litter decomposition in temperate forests was weaker than in tropical forests. Mayer, Ostertag & Cowie (2011) reported that soil animals increased the decomposition rate by about 17% in tropical rainforests. Meanwhile, the soil fauna promoted the release of N and Mn from litter. In the decomposition of coniferous litter, soil fauna increased N and Mn release from litter by 33% and 21.5%, respectively (Kampichler & Bruckner, 2009). In addition, the structure and activity of microbial communities can be modified by soil fauna (Hättenschwiler, Tiunov & Scheu, 2005). A study found that terrestrial isopods could improve soil nutrient availability and accelerate litter decomposition (Yang, Shao & Li, 2020). Therefore, analyzing the relationship between soil fauna, climate and litter quality is of great significance in achieving a deeper insight into the nutrient cycles in the forest ecosystem.

García-Palacios et al. (2013) and Zhang et al. (2015) found that the litter decomposition rates in global ecosystems were reduced by 35% when the soil fauna was absent. Global studies have advanced our understanding of the impacts of soil biota on litter decomposition. However, forest ecosystems in China contain a wide range of climate types and dominant tree species (Cao et al., 2006; Wang, Ruan & Wang, 2009; Wang et al., 2010). In 2001, Chinese forest cover reached 133.7 million’ hectares (Fang et al., 2001). Chinese forest ecosystems cover almost all major forest communities in the Northern Hemisphere (Zhu et al., 2017). Therefore, it is of great importance to understand the patterns of soil fauna influence on litter decomposition in forest ecosystems to explain the global ecosystem C cycle. In fact, there have been relatively few studies related to the roles of soil fauna in litter decomposition in Chinese forests (Yin et al., 2002; Wang, Ruan & Wang, 2009; Li et al., 2014). Previous studies, carried out at a specific location, as in most cases on Chinese forests, identify only part of the mechanism; a study of a wide range of experiments into litter decomposition at different climates is needed. Moreover, no review article has been published on the status of forest litter decomposition and soil fauna throughout China.

In this contribution, to fill in this knowledge gap, we perform a meta-analysis of the effects of soil fauna on litter decomposition across forests in China. This paper re-analyzes data from experiments on litter decomposition in different forest biomes throughout China, and is aimed at providing a comprehensive analysis of the effects of soil fauna on litter decomposition rates in Chinese forests, by reviewing research data on how climatic factors and litter chemical properties interact with soil fauna to affect the rate of litter decomposition. We tested two hypotheses: (1) the influence of soil fauna on litter decomposition in different forest types at various climates is: tropical forest > subtropical forest > temperate forest; and (2) the effect of soil fauna on decomposition may be related to precipitation, and the concentrations of nitrogen (N), lignin and tannins in the litter.

## Survey Methodology

### Data collection

**Figure 1:**
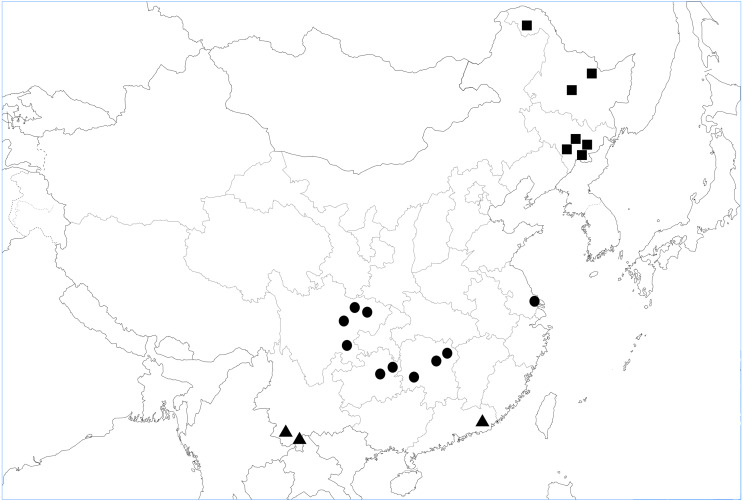
Map showing the location of 20 independent study sites in analysis. ■ Study sites in temperate forests, ▴ study sites in tropical forests, and • study sites in subtropic forests.

We reviewed studies carried out in Chinese forests which evaluated the effects of soil fauna on litter decomposition. Subject words and keywords, including “litter decomposition”, “soil animals”, “soil fauna”, “China” and “forest”, were combined during literature retrieval. A comprehensive literature search was conducted in China National Knowledge Infrastructure (*CNKI*, http://cnki.net/), Google Scholar (https://scholar.google.com/), and Web of Science (https://www.webofscience.com/wos/alldb/basic-search). Collection of the relevant research was made in May 2017, with no restriction for publication date. The primary search obtained 100 papers. Overall, 95% of the studies used litterbags with different mesh sizes to exclude soil animals, whereas the remaining 5% of the studies used chemicals for this purpose. With respect to mesh sizes, fine mesh ranged from 10 to 2,000 µm, with coarser mesh ranging from 500 to 5,000 µm. In our analysis, the key papers needed to follow the following criteria. First, the treatments resulting in soil fauna excluded (or reduced) *vs.* soil fauna present were achieved either by comparing litterbag mesh sizes (fine or coarse) or chemicals (such as naphthalene, including the dose used, and the use of a control treatment). Second, research had to compare litter mass loss between field trials in both the presence and absence of fauna. Third, an important aim of our research was to analyze whether test conditions and experimentally created environment caused different responses of litter to fauna. Among the 100 papers initially selected, we identified 20 suitable papers which satisfied these criteria in the database (Appendix S1). All the 20 studies selected were based on field experiments in tropical, subtropical, or temperate forests in China (Fig. 1). Fauna-free and control soil treatments were included in all 20 studies. The duration of each of the studies included in the database was greater than nine months.

We collected the available climatic data (*e.g.*, mean annual precipitation, mean annual temperature) and information on the initial litter quality, including concentrations of lignin, cellulose, N, phosphorus (P), and C: N ratios from the papers. In addition, we prioritized those papers providing the most detailed information. To assess the effect of the mesh size of litterbags was tested only at two levels, comparing small (10–2,000 µm) and large (850–6,000 µm) mesh sizes.

In the database were also store data on 64 plant species and 120 different species combinations (single species or species mixtures; Appendix S1). Moreover, 120 decomposition constants (*k*) were obtained by calculating litter mass loss in the papers in the database.

Data were extracted directly from the relevant figures or tables in each study, using the GetData Graph Digitizer to obtain the mean, standard deviation, and sample size.

### Data analysis

The data collected from each study were re-analyzed, using the exponential decomposition model (Olson, 1963):

\begin{eqnarray*} \frac{{M}_{t}}{{M}_{0}} ={e}^{-kt} \end{eqnarray*}

where *M*_t_ is dry litter mass at time t (y), *M*_0_ is the initial dry litter mass (at time 0), and *k* is the decomposition rate constant (y^−1^). For studies carried out over long time scales, the single exponential decay model may not give the best estimate of the decay pattern (Berg & Matzner, 1997), but it is the most widely used model in litterbag decomposition studies (Silver & Miya, 2001). The method of soil fauna contribution to litterbag decomposition used was that proposed by Seastedt (1984). Meanwhile, the contribution of soil fauna to litter decomposition was calculated by the difference in litter decomposition constant rates (*k*) between bags in the presence and absence of soil fauna.

We calculated the effect size (ES) of the decomposition rate, using ES = ln (*k*_*e*_ / *k*_*i*_), where *k*_*e*_ is calculated when soil fauna is excluded (or reduced) in litterbags, and *k*_*i*_ when soil fauna is present. ES represents a unit-free index, ranging from − ∞ to + ∞, indicating the magnitude and direction of the influence (Berg & Matzner, 1997). Positive values indicate accelerated litter decomposition, whereas negative ES values indicate slower litter decomposition following exclusion of soil fauna.

If any variables did not approximate to a normal distribution, they were log-transformed to fulfill the assumptions in order to carry out analysis of variance (ANOVA). We compared ES value between chemical *vs.* physical soil fauna exclusion and inclusion methods. Linear regressions and one-way ANOVA were used to test for differences in the relationships between ES and either litter quality or climate parameters. Means were compared using Tukey multiple comparison tests. Simple and stepwise parametric multiple linear regressions were performed to evaluate whether initial litter quality indices and/or climate variables were correlated with ES. Critical significance level was defined as *P* < 0.05. Statistical analyses were performed with SPSS Statistics v.22 (IBM, Armonk, NY, USA).

## Results

**Figure 2:**
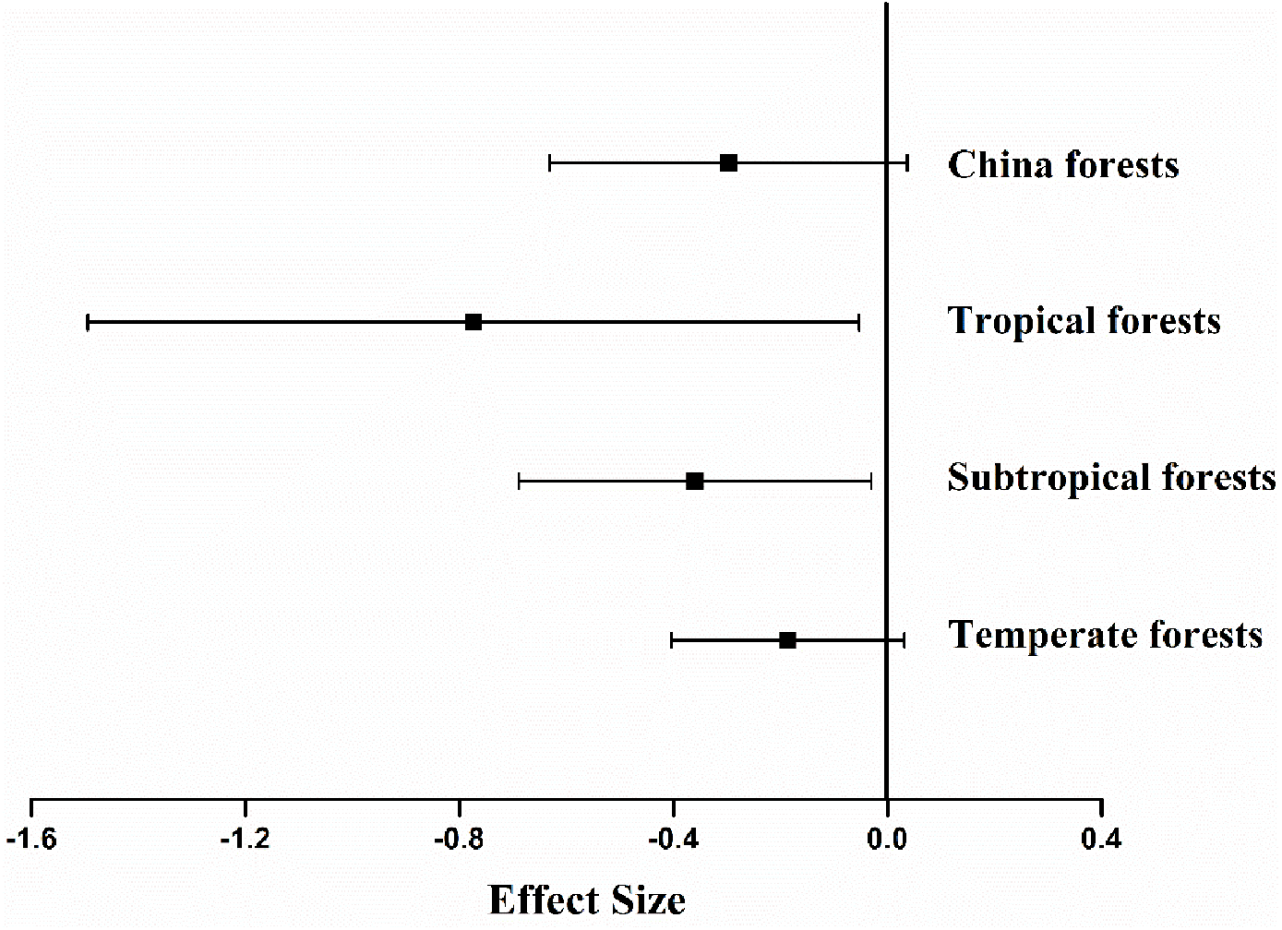
Mean effect size of soil fauna exclusion on litter decomposition rates at nationwide scale (*n*= 126), tropical forests (*n*= 6), subtropical forests (*n*= 57), temperate forests (*n*= 63). The bars around the means are standard deviation. The negative effect size indicates that litter decomposition is slower without soil fauna.

The experimental sites studied spanned 31 degrees of latitude, from 21°N to 52°N, with mean annual temperature (MAT) ranging from −5 °C to 21.5 °C, and mean annual precipitation (MAP) from 428 to 3,100 mm. Other variables monitored included concentrations of N (0.3–3.6%), lignin (8.6–30.5%), P (0.0007–0.34%), cellulose (7.3–25.0%), lignin: N ratio (5.9–95.9), C: N ratio (16.6–172) and N: P ratio (3.6–528.6) of the litter dry weight. The effects of differences in methodology on litter decomposition rates resulted not significantly different (*P* = 0.094). Analysis indicated that mean ES differed among climate types (Fig. 2). The average ES in China was −0.297 (*P* < 0.001, Fig. 2), and the forest litter decomposition rates were reduced by 65% when soil animals were excluded. Among the three types of forest ecosystem, fauna-free soils showed reduced litter decomposition rates to varying degrees, with a reduction of 28% in temperate forests, 47% in subtropical forests, and 200% in tropical forests.

**Figure 3:**
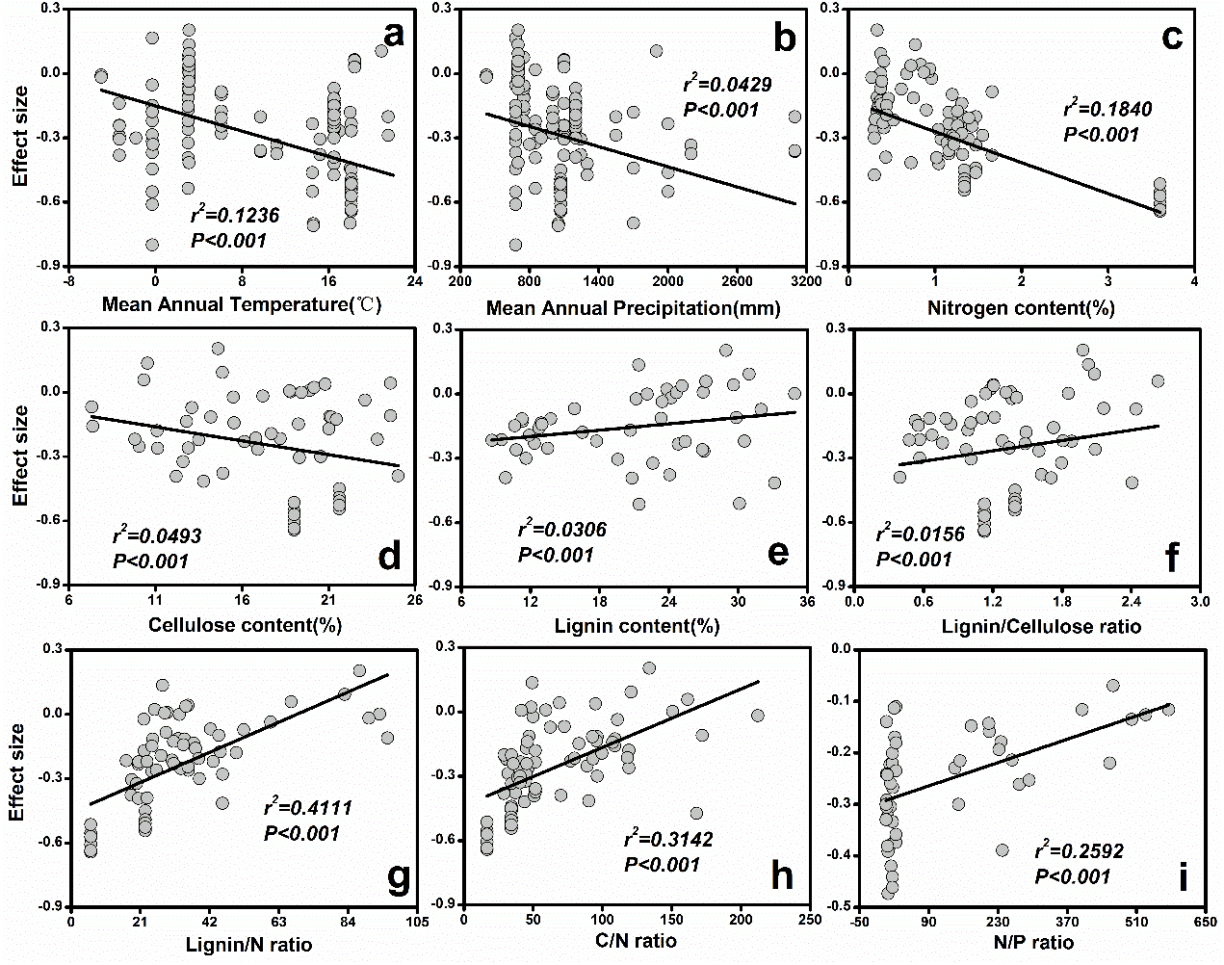
The relationships between initial litter quality and ES. (A) Mean annual temperature; (B) Mean annual precipitation; (C) Nitrogen content (%); (D) Cellulose content (%); (E) Lignin content (%); (F) Lignin/Cellulose ratio; (G) Lignin/N ratio; (H) C/N ratio; (I) N/P ratio.

The results from the current study indicated that MAP (*r*^2^ = 0.12, *P* < 0.001) and MAT (*r*^2^ = 0.04, *P* < 0.001) explained most of the variation in soil fauna effects (Fig. 3). Specifically, ES decreased with increasing MAT (Fig. 3A) or MAP (Fig. 3B). Additionally, initial chemical properties of litter quality had different relative effects on soil fauna ES. The ES was reduced with increasing nitrogen (*r*^2^ = 0.18, *P* < 0.001) and cellulose concentrations (*r*^2^ = 0.05, *P* < 0.001; Fig. 3). On the other hand, lignin concentration (*r*^2^ = 0.03, *P* < 0.001), C: N ratio (*r*^2^ = 0.31, *P* < 0.001), N: P ratio (*r*^2^ = 0.26, *P* < 0.001) and lignin: N ratio (*r*^2^ = 0.41, *P* < 0.001) showed significant positive relationships with ES (Fig. 3).

Considering all the litter quality parameters, the best-fit multiple regression proved to be most suitable for describing the litter quality model. Combining N content and C: N ratio had a significant effect on ES (*r*^2^ = 0.46, *P* < 0.001). The combination of climatic and litter quality factors generated the strongest relationships with ES (Table 1). In particular, the predictive power of ES was greatest (*r*^2^ = 0.54, *P* < 0.001) when the effects of N concentration, C: N rate and MAP were combined (Table 1).

## Discussion

### Soil fauna has a consistently positive impact on litter decomposition rate in Chinese forests.

This meta-analysis indicated that the presence of soil fauna increased forest litter decomposition rates by an average of 65% in Chinese forests. Although there was large variation across regions, all the forest ecosystems in the different biomes examined showed a consistent positive effect of soil fauna on litter decomposition rates. The effect of soil fauna was greatest in tropical forests and lowest in cold temperate forest ecosystems, where decomposition rate was also constrained by temperature, moisture, and/or litter quality.

Mean annual temperature (MAT) and mean annual precipitation (MAP) were the most critical climatic variables identified in many studies (Yin et al., 2019). The observed results indicate that the effect of soil fauna on decomposition rate increased with increasing precipitation and temperature, a finding which is consistent with that of previous research (García-Palacios et al., 2013). This can be related to the effects of climatic factors, especially precipitation (González & Seastedt, 2001) and temperature (Heneghan et al., 1999), on the activities of the soil fauna.

Liu et al. (2019) showed that MAT was the primary abiotic factor affecting the contribution of soil fauna to C release in Chinese alpine forest ecosystems. Furthermore, only forest ecosystems were included in this study. It was found that MAP has fewer influences over ES than does MAT. This may be due to the low number of studies we examined in our meta-analysis. In the current analysis, soil fauna may be most active at sites with high MAP, whereas higher MAP speeds up the leaching to release process (Yu et al., 2019). Leaching affects the physical structure of litter, which is related to direct feeding by soil fauna. In particular, the MAP in temperate forests is lower than in tropical and subtropical forests (S1). In southwestern of China, higher precipitation leads to an increase in soil faunal diversity (Tan et al., 2020), so that soil fauna effects are increased by MAP. Meanwhile, precipitation directly affects soil physical structure, stimulating soil fauna to decompose litter. An interesting study suggests that litter quality may be transmitted from soil structure by protists, nematodes, and microarthropods (Elliott & Coleman, 1988; Erktan, Or & Scheu, 2020). Dry and wet cycles cause cracks in the soil that increase pore connectivity (Koestel, 2020) and promote litter quality transfer to deeper soil depths, contributing to the nutrient cycling process to a significant degree. Hence, the MAP directly affects litter decomposition by soil fauna to a considerable degree. On the other hand, large- and medium-sized soil fauna would shred plant litter, and the number of microorganisms in the ecosystem would change as a consequence of the generation of litter debris and release of soil fauna metabolites. Simultaneously, bacteria and fungi would be attracted to the litter, stimulating even more microbial litter decomposition (Lukumbuzya, Fyles & Cote, 1994). Temperate regions are relatively drier and colder than other biomes, with such conditions negatively affecting the decomposition rate of litter by soil fauna (Aerts, 2006).

**Table 1:** Best-fit multiple regressions of litter decomposition with litter quality and climatic factors across all studies.

Model equation	*d.f.*	*r* ^2^	*r*	*P*
Environmental climatic variables only: ES = −0.150–0.015MAT	125	0.131	0.361	<0.0001
Leaf quality variables only: ES = 0.133–0.002C/N-0.251N	44	0.455	0.675	<0.0001
Combined leaf quality and climate variables: ES = 0.338–0.242N-0.001C/N+0.0003MAP	44	0.538	0.734	<0.0001

### Relationship between substrate quality and soil fauna ES

The results showed that the contribution to decomposition of litter by soil fauna depended to some extent on litter quality (Table 1, Fig. 2). Variables such as concentrations of N (Hunt et al., 1988) and lignin (Meentemeyer, 1978), as well as lignin: N ratio (Ostertag & Hobbie, 1999), have been suggested as predictors of plant litter decomposition rates (Melillo, Aber & Muratore, 1982). These factors may also be closely related to soil fauna activity. When N and cellulose concentrations in the litter quality were relatively high, the decomposition rate was more affected by exclusion of soil fauna. Research suggests that leaf decomposition is positively correlated with N concentration (Hunt et al., 1988; Guo et al., 2008). The soil fauna and microorganisms obtain their N supplies from the litter as an input to improve the decomposition process, and a low N concentration will slow down the decomposition rate (Aerts & De Caluwe, 1997).

Lignin is generally considered to be a substance recalcitrant to decomposition. It mainly strengthens and waterproofs cell walls in woody plant tissues. Soil fauna will become less likely to feed on resources with a high lignin concentration. On one hand, the decomposition rate of plant litter is negatively related to the resistance of lignin to enzymes (Wang et al., 2018a; Wang et al., 2018b). On the other hand, the physical structure of lignin is also affected when soil fauna feeds on the litter (Yue et al., 2016). In Fig. 3, the ES was negatively correlated with lignin concentration (Guo et al., 2008). This may be due to the soil fauna destroying the physical structure of lignin. In Chinese alpine forest-tundra ecotone, Wang et al. (2018a) and Wang et al. (2018b) found that soil fauna promoted lignin degradation rates during litter decomposition. Thus, the soil fauna effect is greater for recalcitrant litter (Fig. 3). Zhang et al. (2015) also suggested that soil fauna has a greater effect on those forms of lignin that are more readily decompostable.

The C: N ratio of the litter was also considered to be an important factor in determining decomposition rate. The importance of litter quality parameters, such as C: N ratios, in governing decomposition rate was recognized as early as 1916 (Jensen, 1929). With high C: N ratios, litter is more difficult to break down (Wickings et al., 2012; Hobbie, 2015). The soil fauna had a significant negative impact on the loss of litter mass with a high C: N ratio (*Hattenschwiler et al., 2005*). The current study also found that an increase in C: N ratio weakened the impact of soil animals on the litter decomposition rate (Fig. 3, Table 1). Coq et al. (2010) suggested that soil fauna may ignore litter with higher C: N ratios when they feed. Yang, Shao & Li (2020) reported that the terrestrial isopods preferred litter with low C: N. Therefore, the higher the C: N ratio of the litter, the lower the influence of soil fauna on litter decomposition.

### Interaction between the litter decomposition environment and soil fauna

The relationship among soil fauna, litter quality, and climatic factors is complex. In many single-site studies, their relationship is difficult to assess. García-Palacios et al. (2013) documented that climate may lead to changes in litter quality in some biomes and thus affect litter decomposition by soil fauna. In southwestern China, soil fauna accelerated the N and P release effect significantly (Yan et al., 2019). Decomposition of low-quality litter was faster in a P-rich environment (Feng, Xue & Chen, 2018; Ochoa-Hueso et al., 2019). In our multi-site meta-analysis, the combination of litter N concentration, MAP and N: P ratio provided the best model of litter substrate quality for predicting forest litter decomposition rates in China (Table 1). Peña Peña & Irmler (2016) found that changes in relative humidity mainly affected litter decomposition rates by modifying the presence and activity of soil biota, while faunal and microbial communities were affected by the litter quality and the climate. Frouz et al. (2015) also explained that soil fauna significantly increased litter loss but had no significant effect on C mineralization. Decomposition and mineralization are very complex processes that are inextricably linked with the physical soil environment and the soil faunal community structure. The microarthropod community plays a key role in soil mineralization (Sulkava, Huhta & Laakso, 1996). Furthermore, there are interactions between the environment, litter, and fauna. Establishing a bridge among these three factors would enable us to predict trends in local ecosystems.

These factors have been shown in many studies to exhibit different degrees of influence on litter decomposition (Lin et al., 2019; Huang et al., 2020). However, the data from published studies were limited; for example, there were fewer samples from tropical regions. A better understanding is needed of the relationship between soil fauna and these factors, requiring the study of more research sites and more species to achieve a comprehensive analysis.

### Further research and future research directions

In our study, the concentrations of N, P, lignin, and cellulose in the initial litter material were shown to provide an effective indicator of the influence of substrate quality on decomposition rate. However, these factors may not necessarily be the best indicators for predicting soil fauna effects, and other factors in the litter, such as concentrations of magnesium and tannins, should be considered in future studies. Tannins are widely considered to be defensive materials for plant protection to reduce consumption of living plant tissues (Coq et al., 2010). They are toxic substances that are unfavorable, when consumed, for the growth of soil fauna, because the interaction with tannins makes the litter protein less bioavailable to the soil fauna. Plants, such as *Anemone nemorosa* (an indicator species of ancient temperate forests), contain glycosides which increase the water-solubility of compounds such as phenols: Lukianchuk et al. (2017) reported that tannins (examples of phenolics) usually cause toxic reactions in some detritivores. Magnesium is also a necessary element for the development of soil biota. High concentrations of magnesium in litter probably promote litter decomposition by soil fauna (Rosenthal & Berenbaum, 2012).

We concluded that relatively few studies on soil fauna diversity and litter decomposition were published, making discussion of the limited results difficult. Recent research findings have filled a gap in knowledge about soil fauna biodiversity and abundance. Yang et al. (2021) showed that soil faunal orders, such as the Araneae, Acarina, Collembola, Isoptera, and Coleoptera (including larvae), accounted for 37.5% of the soil fauna in semi-arid continental climates. A study of litter N concentration on soil fauna abundance and litter decomposition rates found that members of the superorders Isotomidae and Parasitiformes were present at high densities in temperate forests. And the N addition treatment significantly increased soil faunal diversity and had a positive effect on the late stage of apoplastic decomposition (Liu et al., 2021).

Moreover, there is no consensus on the impact of biodiversity on litter decomposition rate. Many studies have found that the diversity of soil fauna is positively correlated with litter decomposition rate (González & Seastedt, 2001). In general, the greater the number and types of soil animals, the faster litter will decompose. Yet differences in litter decomposition rates cannot be explained solely by soil fauna diversity and richness (Heneghan et al., 1999; González & Seastedt, 2001). In structural equation modeling, litter decomposition rate is affected by the nutrients leaching from the litter. Changes in the soil fauna abundance, especially with respect to omnivores and detritivores, would not be expected to affect litter decomposition rate (Wang et al., 2021). Decomposition is a very complex process, and the diversity and richness of the fauna is greatly affected by environmental factors, which, in turn, impact upon the litter decomposition rate. Furthermore, the predator–prey relationship among soil organisms is another factor with an unknown impact on it.

In order to control the soil fauna which participated in litter decomposition, researchers used different-sized mesh to restrict the entry and exit of soil animals of different sizes, although some studies included in the database of this study chose to use chemical reagents to suppress the diversity and abundance of soil fauna species in the study area (Kampichler & Bruckner, 2009). But the litterbag and chemical inhibition methods may reduce the accuracy of measuring litter decomposition rates and introduce additional unknown changes to some extent (García-Palacios et al., 2013). Researchers in this discipline have proposed alternative strategies to control the soil fauna in decomposition studies. Makkonen et al. (2012) suggested that field microcosms could be a viable alternative to litterbags. For example, PVC rings have been used to generate field microcosms, a method that obviates the loss of some litter due to leakage through the mesh of litterbags, and prevents destruction of biotic and abiotic elements of the environment by chemical agents. For future studies on the litter decomposition rate by soil fauna, such experimental field microcosm should be considered as an alternative to previously adopted methodologies.

Although much research has been carried out on soil fauna and litter decomposition (Lin et al., 2019), many issues still need to be addressed in future studies, such as the relative roles of microorganisms and soil fauna in litter decomposition. Understanding how soil fauna influence litter decomposition should be closely linked to the involvment of researchers from other subdisciplines in the future, in addition to making improvements in the study methods being used.

## Conclusions

Our meta-analysis shows that the combination of litter quality and climatic parameters can prove to be a good predictor of the contribution of soil fauna to litter decomposition rates in forests in China. The contribution of soil fauna to decomposition plays a key role in soil carbon and nutrient cycling. The results highlighted the effects of climatic factors, such as annual mean temperature and rainfalls, and litter quality parameters, such as N and lignin concentrations and C: N ratio, on the impact of soil fauna on litter decomposition. Understanding the effect of soil fauna on litter decomposition rate will improve our ability to predict global carbon dynamics. In Chinese forest ecosystems, previous studies have largely ignored the impact of soil fauna diversity on litter decomposition. The results from the current meta-analysis should be combined with litter quality and climatic factors parameters to improve our understanding of how soil fauna can influence rates of litter decay at broad spatial scales.

## Supplemental Information

10.7717/peerj.12747/supp-1 Supplemental Information 1 PRISMA checklist

10.7717/peerj.12747/supp-2 Supplemental Information 2 Appendix

## Additional Information and Declarations

### Data Availability

The following information was supplied regarding data availability: The references, case studies and descriptors used in the analysis in this paper are available as a Supplemental File.
