# Supplementary material for: Effects of soil fauna on litter decomposition in Chinese forests: a meta-analysis
Source: PeerJ. 2022 Jan 10;10:e12747. doi: 10.7717/peerj.12747 (PMC8757372; doi:10.7717/peerj.12747)
Supplement: Supplemental Information 2 [file peerj-10-12747-s002.docx]

S1. List of references, case studies and descriptors used in the analysis. Mixture represents litterbags with more than one species litter.

| References | Case study | Plant  species | Method | Fauna excluded mesh  Size(μm ) | Fauna accessed mesh  Size(μm ) | Harvests | Study length  (year) | Effect size | Climate type | Latitude  (North) | Longitude  (East) |
| --- | --- | --- | --- | --- | --- | --- | --- | --- | --- | --- | --- |
| Xiong *et al.* (2005) |  | Mixture | litterbag | 10 | 2000 | 1 | 3 | 0.1059 | Tropic | 23°09′ | 112°30′ |
| Yang *et al.* (2009) |  | Mixture | litterbag | 150 | 2000 | 3 | 1 | -0.2013 | Tropic | 21°56′ | 101°11′ |
| Yang *et al.* (2009) |  | Mixture | litterbag | 150 | 2000 | 3 | 1 | -0.2874 | Tropic | 21°56′ | 101°11′ |
| Yang *et al.* (2009) |  | Mixture | litterbag | 150 | 2000 | 3 | 1 | -1.4580 | Tropic | 21°56′ | 101°11′ |
| Yang *et al.* (2006) |  | Mixture | litterbag | 150 | 2000 | 2 | 1 | -1.3715 | Tropic | 21°41′ | 101°25′ |
| Yang *et al.* (2006) |  | Mixture | litterbag | 150 | 2000 | 2 | 1 | -1.4321 | Tropic | 21°41′ | 101°25′ |
| Wang *et al.* (2009) |  | *Castanopsis carlesii* | naphthalene | 1000 | 1000 | 4 | 1 | -0.6969 | Subtropics | 27°33′ | 117°27′ |
| Wang *et al.* (2009) |  | *Castanopsis carlesii* | naphthalene | 1000 | 1000 | 4 | 1 | -0.5495 | Subtropics | 27°33′ | 117°27′ |
| Wang *et al.* (2009) |  | *Castanopsis carlesii* | naphthalene | 1000 | 1000 | 4 | 1 | -0.3341 | Subtropics | 27°33′ | 117°27′ |
| Wang *et al.* (2009) |  | *Castanopsis carlesii* | naphthalene | 1000 | 1000 | 4 | 1 | -0.3629 | Subtropics | 27°33′ | 117°27′ |
| Wang *et al.* (2010) |  | *Castanopsis carlesii* | litterbag | 100 | 5000 | 8 | 1 | -0.4409 | Subtropics | 27°33′ | 117°27′ |
| Wang *et al.* (2010) |  | *Castanopsis carlesii* | litterbag | 100 | 5000 | 8 | 1 | -0.4613 | Subtropics | 27°33′ | 117°27′ |
| Wang *et al.* (2010) |  | *Castanopsis carlesii* | litterbag | 100 | 5000 | 8 | 1 | -0.3337 | Subtropics | 27°33′ | 117°27′ |
| Wang *et al.* (2010) |  | *Castanopsis carlesii* | litterbag | 100 | 5000 | 8 | 1 | -0.2007 | Subtropics | 27°33′ | 117°27′ |
| Wang *et al.* (2010) |  | *Pinus taiwanensis* | litterbag | 100 | 5000 | 8 | 1 | -0.1804 | Subtropics | 27°33′ | 117°27′ |
| Wang *et al.* (2010) |  | *Pinus taiwanensis* | litterbag | 100 | 5000 | 8 | 1 | -0.2344 | Subtropics | 27°33′ | 117°27′ |
| Wang *et al.* (2010) |  | *Pinus taiwanensis* | litterbag | 100 | 5000 | 8 | 1 | -0.3737 | Subtropics | 27°33′ | 117°27′ |
| Wang *et al.* (2010) |  | *Pinus taiwanensis* | litterbag | 100 | 5000 | 8 | 1 | -0.3584 | Subtropics | 27°33′ | 117°27′ |
| Cui *et al.*(2012) |  | *Cunninghamia lanceolata* | field microcosm | 2000 | 4000 | 2 | 0.3 | -0.4199 | Subtropics | 26°50' | 109°36' |
| Cui *et al.*(2012) |  | *Castanopsis fargesii* | field microcosm | 2000 | 4000 | 2 | 0.3 | -0.4733 | Subtropics | 26°50' | 109°36' |
| Wang *et al.*(2013) |  | *Pinus massoniana* | litterbag | 40 | 3000 | 4 | 1 | -0.2334 | Subtropics | 28°34 | 104°32′ |
| Wang *et al.*(2013) |  | *Cinnamomum camphora* | litterbag | 40 | 3000 | 4 | 1 | -0.2671 | Subtropics | 28°34 | 104°32′ |
| Wang *et al.*(2013) |  | *Cryptomeria fortunei* | litterbag | 40 | 3000 | 4 | 1 | -0.3046 | Temperate | 31°01′ | 103°34′ |
| Wang *et al.*(2013) |  | *Quercus acutissima* | litterbag | 40 | 3000 | 4 | 1 | -0.3774 | Temperate | 31°01′ | 103°34′ |
| Liu *et al.*(2013) |  | *Sabina saltuaria* | litterbag | 40 | 3000 | 4 | 1 | -0.3916 | Temperate | 31°14′ | 102°53′ |
| Liu *et al.*(2013) |  | *Salix paraplesia* | litterbag | 40 | 3000 | 4 | 1 | -0.2219 | Temperate | 31°14′ | 102°53′ |
| Liu *et al.*(2013) |  | *Abies faxoniana* | litterbag | 40 | 3000 | 4 | 1 | -0.3235 | Temperate | 31°14′ | 102°53′ |
| Liu *et al.*(2013) |  | *Betula albosinensis* | litterbag | 40 | 3000 | 4 | 1 | -0.2594 | Temperate | 31°14′ | 102°53′ |
| Li *et al.*(2013) |  | *Eucalyptus grandis* | litterbag | 50 | 3000 | 2 | 1 | -0.2268 | Subtropics | 29°36' | 103°36' |
| Li *et al.*(2013) |  | *Alnus formosana* | litterbag | 50 | 3000 | 2 | 1 | -2.2837 | Subtropics | 29°36' | 103°36' |
| Xia *et al.*(2012) |  | *Betula albosinensis* | litterbag | 20 | 3000 | 1 | 0.5 | 0.0175 | Temperate | 31°19' | 102°57' |
| Fan *et al.*(2010) |  | *Zanthoxylum planispinum* | litterbag | 50 | 850 | 5 | 0.7 | 0.0393 | Subtropics | 25°39′ | 105°36′ |
| Fan *et al.*(2010) |  | *Zanthoxylum planispinum* | litterbag | 50 | 850 | 5 | 0.7 | 0.0614 | Subtropics | 25°39′ | 105°36′ |
| Fan *et al.*(2010) |  | *Zanthoxylum planispinum* | litterbag | 50 | 850 | 5 | 0.7 | 0.0650 | Subtropics | 25°39′ | 105°36′ |
| Fan *et al.*(2010) |  | *Zanthoxylum planispinum* | litterbag | 50 | 850 | 5 | 0.7 | 0.0611 | Subtropics | 25°39′ | 105°36′ |
| Fan *et al.*(2010) |  | *Zanthoxylum planispinum* | litterbag | 50 | 850 | 5 | 0.7 | 0.0309 | Subtropics | 25°39′ | 105°36′ |
| Fan *et al.*(2014) |  | Mixture | litterbag | 10 | 5000 | 2 | 1 | -0.6997 | Temperate | 32°52′ | 120°49′ |
| Fan *et al.*(2014) |  | *Populus deltoides* | litterbag | 10 | 5000 | 2 | 1 | -0.7093 | Temperate | 32°52′ | 120°49′ |
| Li *et al.*(2016) |  | *Pinus massoniana* | litterbag | 40 | 3000 | 16 | 1 | -0.5310 | Subtropics | 28°11′ | 104°21′ |
| Li *et al.*(2016) |  | *Pinus massoniana* | litterbag | 40 | 3000 | 16 | 1 | -0.5416 | Subtropics | 28°11′ | 104°21′ |
| Li *et al.*(2016) |  | *Pinus massoniana* | litterbag | 40 | 3000 | 16 | 1 | -0.4491 | Subtropics | 28°11′ | 104°21′ |
| Li *et al.*(2016) |  | *Pinus massoniana* | litterbag | 40 | 3000 | 16 | 1 | -0.4926 | Subtropics | 28°11′ | 104°21′ |
| Li *et al.*(2016) |  | *Pinus massoniana* | litterbag | 40 | 3000 | 16 | 1 | -0.4915 | Subtropics | 28°11′ | 104°21′ |
| Li *et al.*(2016) |  | *Pinus massoniana* | litterbag | 40 | 3000 | 16 | 1 | -0.5073 | Subtropics | 28°11′ | 104°21′ |
| Li *et al.*(2016) |  | *Pinus massoniana* | litterbag | 40 | 3000 | 16 | 1 | -0.5435 | Subtropics | 28°11′ | 104°21′ |
| Li *et al.*(2016) |  | *Cinnamomum camphora* | litterbag | 40 | 3000 | 16 | 1 | -0.5266 | Subtropics | 28°11′ | 104°21′ |
| Li *et al.*(2016) |  | *Cinnamomum camphora* | litterbag | 40 | 3000 | 16 | 1 | -0.6395 | Subtropics | 28°11′ | 104°21′ |
| Li *et al.*(2016) |  | *Cinnamomum camphora* | litterbag | 40 | 3000 | 16 | 1 | -0.6419 | Subtropics | 28°11′ | 104°21′ |
| Li *et al.*(2016) |  | *Cinnamomum camphora* | litterbag | 40 | 3000 | 16 | 1 | -0.5547 | Subtropics | 28°11′ | 104°21′ |
| Li *et al.*(2016) |  | *Cinnamomum camphora* | litterbag | 40 | 3000 | 16 | 1 | -0.5732 | Subtropics | 28°11′ | 104°21′ |
| Li *et al.*(2016) |  | *Cinnamomum camphora* | litterbag | 40 | 3000 | 16 | 1 | -0.6061 | Subtropics | 28°11′ | 104°21′ |
| Li *et al.*(2016) |  | *Cinnamomum camphora* | litterbag | 40 | 3000 | 16 | 1 | -0.5137 | Subtropics | 28°11′ | 104°21′ |
| Li *et al.*(2016) |  | *Cinnamomum camphora* | litterbag | 40 | 3000 | 16 | 1 | -0.6350 | Subtropics | 28°11′ | 104°21′ |
| Li *et al.*(2016) |  | *Cinnamomum camphora* | litterbag | 40 | 3000 | 16 | 1 | -0.5701 | Subtropics | 28°11′ | 104°21′ |
| Zhang *et al.*(2016) |  | *Cunninghamia lanceolata* | litterbag | 100 | 2000 | 18 | 1 | -0.1163 | Subtropics | 26°40′ | 109°26′ |
| Zhang *et al.*(2016) |  | *Michelia alba* | litterbag | 100 | 2000 | 18 | 1 | -0.1350 | Subtropics | 26°40′ | 109°26′ |
| Zhang *et al.*(2016) |  | *Michelia macclurei* | litterbag | 100 | 2000 | 18 | 1 | -0.1258 | Subtropics | 26°40′ | 109°26′ |
| Zhang *et al.*(2016) |  | *Osmanthus sp* | litterbag | 100 | 2000 | 18 | 1 | -0.1585 | Subtropics | 26°40′ | 109°26′ |
| Zhang *et al.*(2016) |  | *Phoebe bournei* | litterbag | 100 | 2000 | 18 | 1 | -0.1787 | Subtropics | 26°40′ | 109°26′ |
| Zhang *et al.*(2016) |  | *Symplocos laurina* | litterbag | 100 | 2000 | 18 | 1 | -0.0690 | Subtropics | 26°40′ | 109°26′ |
| Zhang *et al.*(2016) |  | *Cyclobalanopsis glauca* | litterbag | 100 | 2000 | 18 | 1 | -0.2611 | Subtropics | 26°40′ | 109°26′ |
| Zhang *et al.*(2016) |  | *Cinnamomum porrectum* | litterbag | 100 | 2000 | 18 | 1 | -0.1424 | Subtropics | 26°40′ | 109°26′ |
| Zhang *et al.*(2016) |  | *Liquidambar formosana* | litterbag | 100 | 2000 | 18 | 1 | -0.2529 | Subtropics | 26°40′ | 109°26′ |
| Zhang *et al.*(2016) |  | *Machilus Pauhoi* | litterbag | 100 | 2000 | 18 | 1 | -0.3001 | Subtropics | 26°40′ | 109°26′ |
| Zhang *et al.*(2016) |  | *Quercus variabilis* | litterbag | 100 | 2000 | 18 | 1 | -0.2199 | Subtropics | 26°40′ | 109°26′ |
| Zhang *et al.*(2016) |  | *Schima superba* | litterbag | 100 | 2000 | 18 | 1 | -0.1163 | Subtropics | 26°40′ | 109°26′ |
| Zhang *et al.*(2016) |  | *Castanopsis hystri* | litterbag | 100 | 2000 | 18 | 1 | -0.2142 | Subtropics | 26°40′ | 109°26′ |
| Zhang *et al.*(2016) |  | *Magnolia liliflora* | litterbag | 100 | 2000 | 18 | 1 | -0.2293 | Subtropics | 26°40′ | 109°26′ |
| Zhang *et al.*(2016) |  | *Paulownia Sieb.* | litterbag | 100 | 2000 | 18 | 1 | -0.3893 | Subtropics | 26°40′ | 109°26′ |
| Zhang *et al.*(2016) |  | *Quercus laevis* | litterbag | 100 | 2000 | 18 | 1 | -0.2154 | Subtropics | 26°40′ | 109°26′ |
| Zhang *et al.*(2016) |  | *Sorbus folgneri* | litterbag | 100 | 2000 | 18 | 1 | -0.1476 | Subtropics | 26°40′ | 109°26′ |
| Zhang *et al.*(2016) |  | *Sorbus folgneri* | litterbag | 100 | 2000 | 18 | 1 | -0.1931 | Subtropics | 26°40′ | 109°26′ |
| Zhang *et al.*(2013) |  | *Abies nephrolepis* | litterbag | 10 | 1000 | 3 | 0.5 | -0.3001 | Temperate | 42°04' | 128°04' |
| Zhang *et al.*(2013) |  | *Abies nephrolepis* | litterbag | 10 | 1000 | 3 | 0.5 | -0.3295 | Temperate | 42°10' | 128°09' |
| Zhang *et al.*(2013) |  | *Abies nephrolepis* | litterbag | 10 | 1000 | 3 | 0.5 | -0.5354 | Temperate | 42°10' | 128°09' |
| Jiang *et al.*(2013) |  | *Pinus koraiensis* | litterbag | 10 | 4000 | 8 | 2 | -0.1104 | Temperate | 42°24′ | 128°5′ |
| Jiang *et al.*(2013) |  | *Fraxinus mandshurica* | litterbag | 10 | 4000 | 8 | 2 | -0.1693 | Temperate | 42°24′ | 128°5′ |
| Jiang *et al.*(2013) |  | *Tilia amurensis* | litterbag | 10 | 4000 | 8 | 2 | -0.2196 | Temperate | 42°24′ | 128°5′ |
| Jiang *et al.*(2013) |  | *Acer mono* | litterbag | 10 | 4000 | 8 | 2 | -0.1130 | Temperate | 42°24′ | 128°5′ |
| Jiang *et al.*(2013) |  | Mixture | litterbag | 10 | 4000 | 8 | 2 | -0.0697 | Temperate | 42°24′ | 128°5′ |
| Jiang *et al.*(2013) |  | Mixture | litterbag | 10 | 4000 | 8 | 2 | 0.0755 | Temperate | 42°24′ | 128°5′ |
| Jiang *et al.*(2013) |  | Mixture | litterbag | 10 | 4000 | 8 | 2 | -0.0574 | Temperate | 42°24′ | 128°5′ |
| Jiang *et al.*(2013) |  | Mixture | litterbag | 10 | 4000 | 8 | 2 | -0.1525 | Temperate | 42°24′ | 128°5′ |
| Li *et al.*(2014) |  | *Betula ermanii* | litterbag | 10 | 4000 | 6 | 1 | -0.0972 | Temperate | 42°03' | 128°03' |
| Li *et al.*(2014) |  | *Rhodendron aureu* | litterbag | 10 | 4000 | 6 | 1 | -0.2796 | Temperate | 42°03' | 128°03' |
| Li *et al.*(2014) |  | *Parasenecio komarovianus* | litterbag | 10 | 4000 | 6 | 1 | -0.0843 | Temperate | 42°03' | 128°03' |
| Li *et al.*(2014) |  | Mixture | litterbag | 10 | 4000 | 6 | 1 | -0.1744 | Temperate | 42°03' | 128°03' |
| Li *et al.*(2014) |  | Mixture | litterbag | 10 | 4000 | 6 | 1 | -0.2451 | Temperate | 42°03' | 128°03' |
| Li *et al.*(2014) |  | Mixture | litterbag | 10 | 4000 | 6 | 1 | -0.2066 | Temperate | 42°03' | 128°03' |
| Yin *et al.*(2002) |  | *Fraxinus mandshurica* | litterbag | 50 | 3000 | 12 | 3 | -0.3153 | Temperate | 47°10′ | 128°53′ |
| Yin *et al.*(2002) |  | *Phellodondron amuriensis* | litterbag | 50 | 3000 | 12 | 3 | -0.4452 | Temperate | 47°10′ | 128°53′ |
| Yin *et al.*(2002) |  | *Juglans mandshurica* | litterbag | 50 | 3000 | 12 | 3 | -0.1476 | Temperate | 47°10′ | 128°53′ |
| Yin *et al.*(2002) |  | *Tilia amurensis* | litterbag | 50 | 3000 | 12 | 3 | -0.6102 | Temperate | 47°10′ | 128°53′ |
| Yin *et al.*(2002) |  | *Acer mono* | litterbag | 50 | 3000 | 12 | 3 | -0.7982 | Temperate | 47°10′ | 128°53′ |
| Yin *et al.*(2002) |  | *Pinus koraiensis* | litterbag | 50 | 3000 | 12 | 3 | -0.0523 | Temperate | 47°10′ | 128°53′ |
| Yin *et al.*(2002) |  | *Ulmus laciniata* | litterbag | 50 | 3000 | 12 | 3 | -0.2196 | Temperate | 47°10′ | 128°53′ |
| Yin *et al.*(2002) |  | *Populus ussuriensis* | litterbag | 50 | 3000 | 12 | 3 | -0.3677 | Temperate | 47°10′ | 128°53′ |
| Yin *et al.*(2002) |  | *Betula platyphylla* | litterbag | 50 | 3000 | 12 | 3 | -0.5507 | Temperate | 47°10′ | 128°53′ |
| Yin *et al.*(2002) |  | *Abies nephrolepis* | litterbag | 50 | 3000 | 12 | 3 | 0.1666 | Temperate | 47°10′ | 128°53′ |
| Yin *et al.*(2002) |  | *Larix gmelinii* | litterbag | 50 | 3000 | 12 | 3 | -0.1775 | Temperate | 47°10′ | 128°53′ |
| Yin *et al.*(2002) |  | *Picea koraiensis* | litterbag | 50 | 3000 | 12 | 3 | -0.2856 | Temperate | 47°10′ | 128°53′ |
| Gao *et al.*(2012) |  | Mixture | litterbag | 80 | 6000 | 3 | 2.5 | -0.0158 | Temperate | 52°09′ | 123°13′ |
| Gao *et al.*(2012) |  | Mixture | litterbag | 80 | 6000 | 3 | 2.5 | -0.0067 | Temperate | 52°09′ | 123°13′ |
| Gao *et al.*(2012) |  | Mixture | litterbag | 80 | 6000 | 3 | 2.5 | -0.0167 | Temperate | 52°09′ | 123°13′ |
| Accepted |  | *Populus davidiana* | field microcosm | 50 | 5000 | 16 | 1 | 0.1352 | Temperate | 45°16′ | 127°34′ |
| Accepted |  | *Betula platyphylla* | field microcosm | 50 | 5000 | 16 | 1 | 0.2035 | Temperate | 45°16′ | 127°34′ |
| Accepted |  | *Sorbus alnifolia* | field microcosm | 50 | 5000 | 16 | 1 | 0.0013 | Temperate | 45°16′ | 127°34′ |
| Accepted |  | *Ulmus davidiana* | field microcosm | 50 | 5000 | 16 | 1 | -0.0716 | Temperate | 45°16′ | 127°34′ |
| Accepted |  | *Acer mono* | field microcosm | 50 | 5000 | 16 | 1 | 0.0421 | Temperate | 45°16′ | 127°34′ |
| Accepted |  | *Phellodendron amurense* | field microcosm | 50 | 5000 | 16 | 1 | -0.0227 | Temperate | 45°16′ | 127°34′ |
| Accepted |  | *Tilia mandshurica* | field microcosm | 50 | 5000 | 16 | 1 | 0.0082 | Temperate | 45°16′ | 127°34′ |
| Accepted |  | *Tilia amurensis* | field microcosm | 50 | 5000 | 16 | 1 | 0.0222 | Temperate | 45°16′ | 127°34′ |
| Accepted |  | *Quercus mongolica* | field microcosm | 50 | 5000 | 16 | 1 | 0.0923 | Temperate | 45°16′ | 127°34′ |
| Accepted |  | *Acer tegmentosum* | field microcosm | 50 | 5000 | 16 | 1 | -0.0008 | Temperate | 45°16′ | 127°34′ |
| Accepted |  | *Amygdalus davidiana* | field microcosm | 50 | 5000 | 16 | 1 | -0.4156 | Temperate | 45°16′ | 127°34′ |
| Accepted |  | *Larix gmelinii* | field microcosm | 50 | 5000 | 16 | 1 | -0.0174 | Temperate | 45°16′ | 127°34′ |
| Accepted |  | *Fraxinus mandshurica* | field microcosm | 50 | 5000 | 16 | 1 | 0.0067 | Temperate | 45°16′ | 127°34′ |
| Accepted |  | *Juglans mandshurica* | field microcosm | 50 | 5000 | 16 | 1 | 0.0372 | Temperate | 45°16′ | 127°34′ |
| Accepted |  | *Pinus sylvestris* | field microcosm | 50 | 5000 | 16 | 1 | 0.0581 | Temperate | 45°16′ | 127°34′ |
| Accepted |  | *Pinus koraiensis* | field microcosm | 50 | 5000 | 16 | 1 | -0.0368 | Temperate | 45°16′ | 127°34′ |

References in the main article：

[1] Xiong, Y.; Liu, Q.; Chen, H.; Peng, S. Leaf litter decomposition of monsoon evergreen broadleaved forest and dynamics and diversity of soil fauna community in Dinghu Mountain. *Chin. J. Ecol.* **2005**, *24*, 1120-1126.

[2] Zhang, R.; Sun, Z.; Wang, C.; Ge, Y., et al. Eco-process of leaf litter decomposition in tropical rain forest in Xishuangbanna, China.Ⅰ. decomposition dynamic of mixed leaf litters. *J. Plant. Ecol.* **2006**, *30*, 780-790.

[3] Wang, S.; Ruan, H.; Wang, B. Effects of soil microarthropods on plant litter decomposition across an elevation gradient in the Wuyi Mountains. *Soil. Biol. Biochem.*, **2009**, *41*, 891-897.

[4] Wang, S.; Ruan, H.; Han, Y. Effects of microclimate, litter type, and mesh size on leaf litter decomposition along an elevation gradient in the Wuyi Mountains, China. *Ecol. Res.* **2010**, *25*, 1113-1120.

[5] Yang, X.; Zou, X. Soil fauna and leaf litter decomposition in tropical rain forest in Xishuangbanna, SW China: effects of mesh size of litterbags. *J. Plant. Ecol*. **2006**, *30*, 791-801.

[6] Cui, Y.; Wang, S.; Yu, X.; Yan, S. Effects of forest soil fauna on early-stage litter decomposition and nutrient release. *Chin. J. Ecol.* **2012**, *31*, 2709-2715.

[7] Wang, W.; Yang, W.; Tan, B.; Liu, R.; Wu, F. Contributions of soil fauna to litter decomposition in subtropical evergreen broad-leaved forests in Sichuan basin. *Ecol. Environ. Sci.* **2013**, *22*, 1488-1495.

[8] Liu, R.; Li, W.; Yang, W.; Tan, B.; Wang, W., et al. Contributions of soil fauna to litter decomposition in alpine/subalpine forests. *Chin. J. Appl. Ecol.* **2013**, *24*, 3354-3360.

[9] Li, Y.; Yang, W.; Luo, C.; Wu, F.; Hu, J. Dynamics on soil faunal community during the decomposition of mixed eucalypt and alder litter. *Acta Ecologica Sinica.*, **2013**, *33*, 0159-0167.

[10] Xia, L.; Wu, F.; Yang, W.; Tan, B. Contribution of soil fauna to the mass loss of *Betula albosinensis* leaf litter at early decomposition stage of subalpine forest litter in western Sichuan. *Chin. J. Appl. Ecol.* **2012**, *23*, 301-306.

[11] Fan, Y.; Chen, H.; Xiong, K.; Su, X., et al. The diversity and litter decomposition function of soil fauna community structure in *Zanthoxylun Planispinum* forestland-a case study of the Huajiang Gorge Area, Guizhou province. *Earth environ.*, **2010**, *38*, 314-319.

[12] Fan, H.; Wang, S.; Ruan, H.; Tan, Y., et al. Effect of soil fauna on litter decomposition and its community structure under different land use patterns in coastal region of northern Jiangsu province. *Journal of Nanjing Forestry University*. *38*, 1-7.

[13] Li, X.; Cui, N.; Zhang, J.; Liu, Y., et al. Mass loss of *Pinus massoniana* and *Cinnamomum camphora* leaf litter in forest gaps of different size. *Chin. J. Appl. Environ. Biol.,* **2016**, *22*, 0292-0299.

[14] Wang, Z.; Yin, X.; Zhang, C. Effect of soil fauna communities on decomposition of *Abies nephrolepis* litter in Changbai Mountains. *Scientia silvae sinicae*. **2016**, *52*, 59-67.

[15] Li, X.; Yin, X.; Wang, Z., et al. Interaction between decomposing litter and soil fauna of the *Betula ermanii* forest floor of the Changbai Mountains, China. *Can. J. Forest Res.* **2014**, *44*, 1507-1514.

[16] Jiang, Y. Litter decomposition and functional role of soil fauna in decomposition in a *Pinus koraiensis* mixed broad-leaved forest of Changbai Mountains. *Changchun: Northeast Normal University.* **2013**.

[17] Li, X. Interaction between decomposing litter and soil fauna of the *Betula ermanii* forest floor of the Changbai Mountains, China. *Changchun: Northeast Normal University.* **2014**.

[18] Yin, X.; Zhong, W.; Wang, H.; Chen, P. Decomposition of forest defoliation and role of soil animals in Xiao Hinggan Mountains. *Geogr. Res*. **2002**, *21*, 689-699.

[19] Gao, M.; Li, J.; Zhang, X. Responses of soil fauna structure and leaf litter decomposition to effective microorganism treatments in Da Hinggan Mountains, China. *Chin. Geogr. Sci.* **2012**, *22*, 647-658.

[20] Zan, P.; Sun, T.; Mao, Z. Effects of soil fauna on litter decomposition using ﬁeld microcosms across 16 co-occurring temperate tree species. Accepted.
